# A Rhinocerotid Skull Cooked-to-Death in a 9.2 Ma-Old Ignimbrite Flow of Turkey

**DOI:** 10.1371/journal.pone.0049997

**Published:** 2012-11-21

**Authors:** Pierre-Olivier Antoine, Maeva J. Orliac, Gokhan Atici, Inan Ulusoy, Erdal Sen, H. Evren Çubukçu, Ebru Albayrak, Neşe Oyal, Erkan Aydar, Sevket Sen

**Affiliations:** 1 Institut des Sciences de l’Évolution, UMR-CNRS 5554, CC064, Université Montpellier 2, Montpellier, France; 2 Department of African Zoology, Royal Museum for Central Africa, Tervuren, Belgium; 3 Department of Geology, General Directorate of MTA, Ankara, Turkey; 4 Hacettepe University Department of Geological Engineering, Beytepe, Ankara, Turkey; 5 General Directorate of MTA, Natural History Museum, Balgat, Ankara, Turkey; 6 Centre de Recherche sur la Paléobiodiversité et les Paléoenvironnements, Muséum National d’Histoire Naturelle, CNRS-UMR 7207, Paris, France; Team ‘Evo-Devo of Vertebrate Dentition’, France

## Abstract

**Background:**

Preservation of fossil vertebrates in volcanic rocks is extremely rare. An articulated skull (cranium and mandible) of a rhinoceros was found in a 9.2±0.1 Ma-old ignimbrite of Cappadocia, Central Turkey. The unusual aspect of the preserved hard tissues of the skull (rough bone surface and brittle dentine) allows suspecting a peri-mortem exposure to a heating source.

**Methodology/Principal Findings:**

Here we describe and identify the skull as belonging to the large two-horned rhinocerotine *Ceratotherium neumayri*, well-known in the late Miocene of the Eastern Mediterranean Province. Gross structural features and microscopic changes of hard tissues (bones and teeth) are then monitored and compared to the results of forensic and archaeological studies and experiments focusing on heating effects, in order to reconstruct the hypothetical peri-mortem conditions. Macroscopic and microscopic structural changes on compact bones (canaliculi and lamellae vanished), as well as partial dentine/cementum disintegration, drastic enamel-dentine disjunctions or microscopic cracks affecting all hard dental tissues (enamel, cementum, and dentine) point to continued exposures to temperatures around 400–450°C. Comparison to other cases of preservation of fossil vertebrates within volcanic rocks points unambiguously to some similarity with the 79 AD Plinian eruption of the Vesuvius, in Italy.

**Conclusions/Significance:**

A 9.2±0.1 Ma-old pyroclastic density current, sourced from the Çardak caldera, likely provoked the instant death of the Karacaşar rhino, before the body of the latter experienced severe dehydration (leading to the wide and sustainable opening of the mouth), was then dismembered within the pyroclastic flow of subaerial origin, the skull being separated from the remnant body and baked under a temperature approximating 400°C, then transported northward, rolled, and trapped in disarray into that pyroclastic flow forming the pinkish Kavak-4 ignimbrite ∼30 km North from the upper Miocene vent.

## Introduction

Volcaniclastic ashes often preserve trace fossils and delicate soft-bodied organisms [1], but also trackways and vertebrate footprints, as in Laetoli (Pliocene, Tanzania [2]–[3]. Volcaniclastic surges may also embed and preserve phosphatized vertebrate remains, as observed in the famous Ashfall Fossil Beds (late Miocene of Nebraska, USA [4] or in Akkasdağı (late Miocene, Turkey [5]). Yet, in more proximal pyroclastic flows, reaching up to 1000°C [6], any embedded organic matter would be cooked and decayed [7]. Somewhat “colder” pyroclastic surges, with temperature ranges of 250–600°C as during the 79 AD Vesuvius eruption in Pompeii, Herculaneum, and Oplontis in Italy, have mostly preserved casts of humans, horses, and pets as well as a few skeletal remains [8]. Be as it may, regardless of the concerned period, fossil records of volcanic origin are extremely rare, as they constitute ∼2% of the total amount of bonebeds at Phanerozoic scale [9].

**Figure 1:**
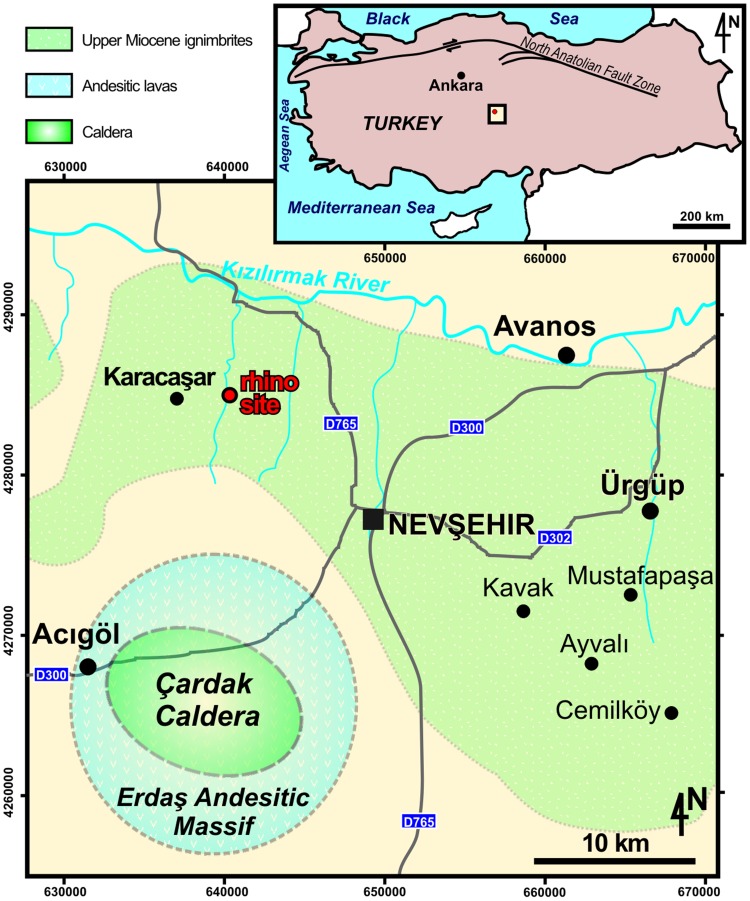
Location map of the Karacaşar rhinocerotine skull, late Miocene of Cappadocia, Central Anatolia, Turkey. The skull (“rhino site”) was unearthed from the 9.2±0.1 Ma-old Kavak-4 subunit of the upper Miocene Kavak ignimbrite, ∼3 km east of Karacaşar. The pyroclastic matrix of the skull probably sourced from the Çardak caldera, ∼30 km more to the South [11]–[13]. Modified after Aydar et al. [12].

**Figure 2:**
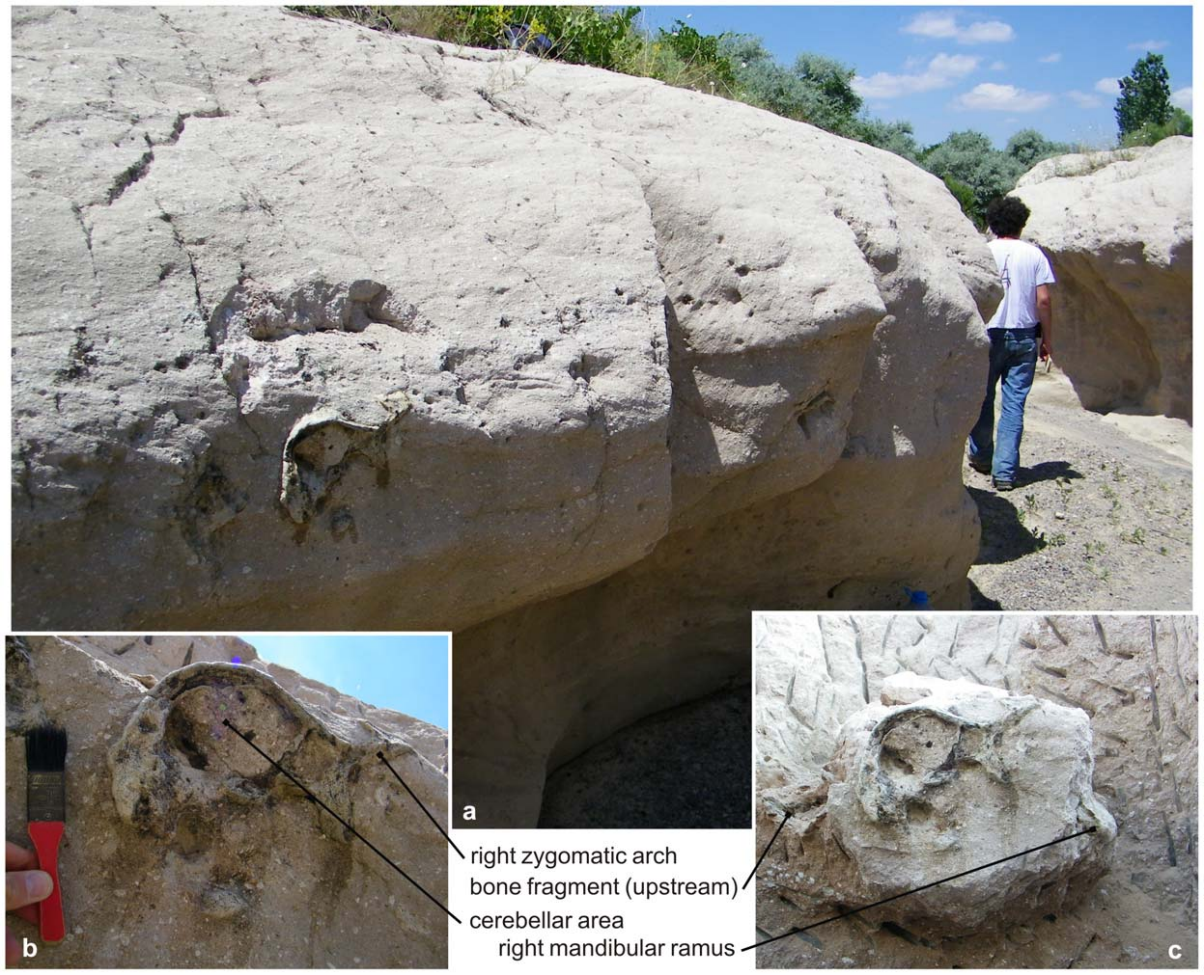
Karacaşar exposure, Cappadocia, Central Anatolia, Turkey. Detail of the exposure (a), showing the cerebellar section of the *Ceratotherium neumayri* cranium cropping out in the bank of the stream (b), and the right *angulus mandibulare* as later appearing during the extraction process (c). Note the presence of bone fragments to the left of the skull (i.e. South to it) and of centimetric whitish pumice clasts both within and around the cerebellar area (b, c).

Here we describe an articulated skull (cranium and mandible) of a large adult rhinocerotid, discovered by the Hacettepe University volcanology team (IU, EŞ, EÇ, and GA) in late June, 2010 while performing geological fieldwork in the Nevşehir-Göreme area, in Cappadocia, Turkey (Figure 1): a coronal-plane section of the cerebellar area was cropping out in a vertical bank of a small stream incised within an ignimbrite flow (N 38°41.819’, E 34°36.811’, 1029 m above sea level; Figure 2). The skull was excavated three days later with the help of a French-Turkish palaeontological team including other authors.

The skull was embedded within the uppermost subunit of the upper Miocene Kavak ignimbrites, termed Kavak-4 [10]–[12]. Its Ar/Ar age is 9.2±0.1 Ma [12]. The concerned ignimbrite subunit consists of a pale pinkish ash-rich matrix with pluricentimetric pumice clasts and scarcer centimetric lithic clasts [11]–[12]. The deposition of the Kavak ignimbrites is considered as being tied to the Çardak caldera, located south of Nevşehir and east to Acıgöl [13], i.e. ∼30 km to the south of Karacaşar (Figure 1). The fabric of the clasts points to a unidirectional channelized flow of subaerial origin (∼N45°W; [12]). Carbonized plant remains were formerly uncovered in that unit [14], but no other vertebrate remain has been recovered from the Kavak ignimbrite to date [12], [14].

After a description, comparison, and identification of the concerned rhino skull, we will discuss the conditions that would have led to the burial and subsequent preservation of such an exceptional fossil.

## Materials and Methods

### Palaeontology

In the descriptive section, capital letters are used for the upper teeth (P, premolar; M, molar), while lower case letters indicate lower teeth (p, m). Except when mentioned, the dimensions are given in mm. The protocol of Guérin [15] is used for measurements.

Dental terminology follows Heissig [16] and Antoine [17]; cranial, mandibular, and dental features described correspond basically to cladistic characters used and listed by Antoine [18]. The suprageneric systematics follows that proposed by Antoine *et al*. [19]–[20].

The specimen described hereunder is stored in the Department of Geological Engineering of the Hacettepe University (HU), in Ankara, Turkey.

### Microscopic Morphological Study

Blocks were extracted from distinct areas of the cranium and mandible, in order to monitor the eventual heating effect on three distinct tissues: cortical bone, enamel, and dentine. The corresponding blocks were mounted on slides and thinned until translumination was considered as satisfactory. Then, they were polished, and observed through a Leica DM EP photomicroscope at X100–X500 magnifications. The microphotographs obtained were digitalized at a resolution of 600 dpi.

The forensic/archeological protocol was adapted from that of Shipman et al. [21] for bones and those of Myers et al. [22], Fereira et al. [23], and Mastrolorenzo *et al*. [8] for hard dental tissues.

Further taxon-dependent comparison was made with unheated teeth of the living Indian rhino *Rhinoceros unicornis*, as control samples.

## Results

### Systematic Palaeontology

Order PERISSODACTYLA Owen, 1848

Superfamily Rhinocerotoidea Owen, 1845

Family Rhinocerotidae Owen, 1845

Subfamily Rhinocerotinae Owen, 1845

Subtribe Rhinocerotina Owen, 1845

Genus *Ceratotherium* Gray, 1867

#### Type species

*Ceratotherium simum* (Burchell, 1817).

### Ceratotherium neumayri *(Osborn, 1900)*

### Description

**Figure 3:**
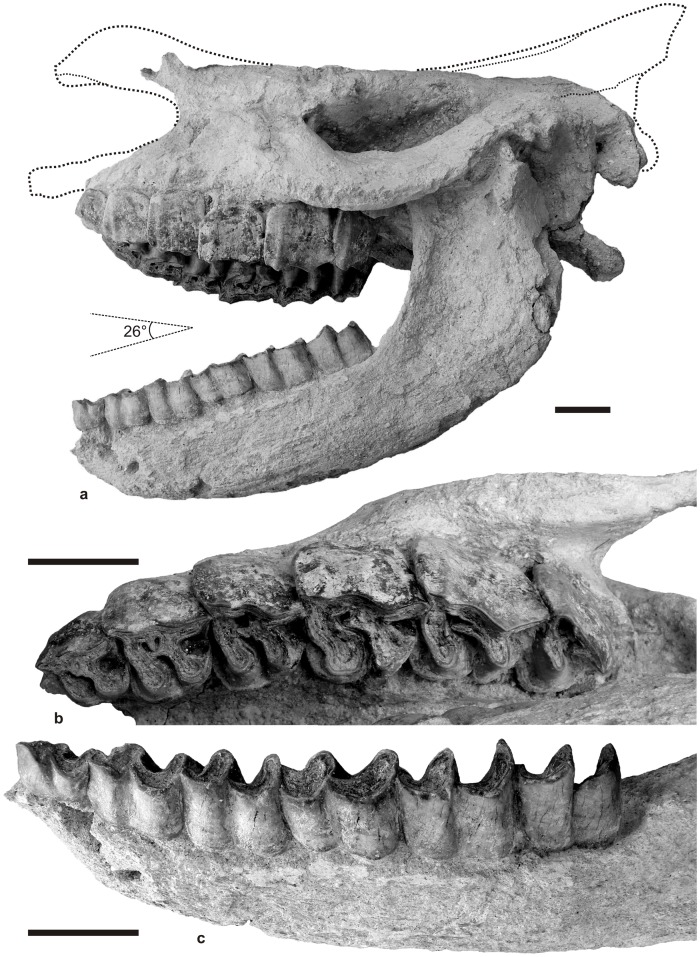
*Ceratotherium neumayri* (Osborn, 1900), Karacaşar (Anatolia, Turkey), late Vallesian (9.2±0.1 Ma). Articulated cranium and mandible (HU-2011-1). a. Left lateral view, with upper/lower cheek teeth angle (ca. 26°) and tentative reconstruction of the lacking parts (maxillae, nasals, parietals, and occipital bone). b. Upper cheek tooth series, with left P2-M3, in occlusal view. c. lower cheek tooth series, with left p2-m3, in labial-occlusal view. The corrugated aspect of the bony surface (3a, 3c) is interpreted as resulting to a long exposure to warm volcaniclastics. Scale bar: 50 mm.

The specimen here described (HU-2011-1) corresponds to the articulated cranium and mandible of a young adult individual, 10–15 years-old (dental stage XI of Hillman-Smith et al. [24]). Both are sub-complete and not deformed, with the temporomandibular joint perfectly articulated in a wide open position (26° angle between occlusal surfaces of tooth rows; Figure 3a).

#### Cranium (Figures 3a, 4b; Table 1)

The adult cranium HU-2011-1 is partly broken: the occipital region is lacking; it was eroded by the small stream the vertical bank of which the specimen was unearthed from, during Holocene times (Figure 2). On the other hand, the premaxillae, as well as most of the nasals and the parietals have also been abraded/destroyed, but most probably peri-mortem (Figure 3a).

**Figure 4:**
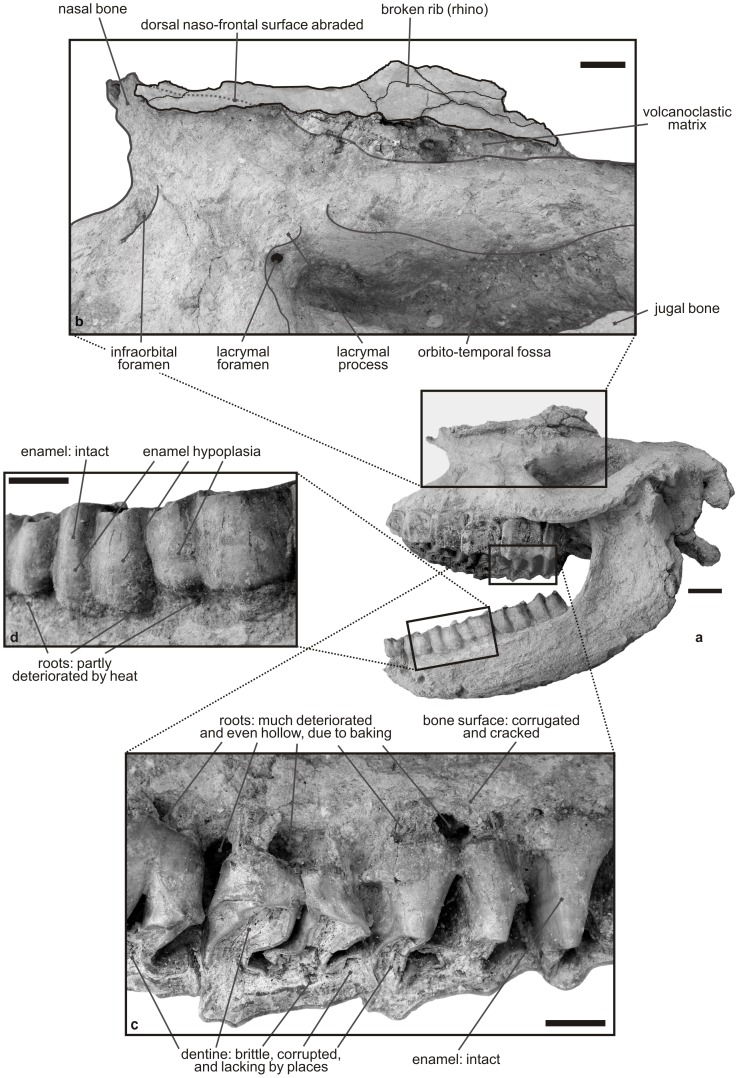
Taphonomical processes involved in the preservation of the Karacaşar rhino. a. general view of the cranium and mandible. b. detail of the dorsal area of the cranium, with a broken rhino rib, trapped ‘upstream’ beside it. c. detail of baked dentine (brittle occlusal surface) and roots (hollow) of right P4-M2. d. labial detail of left p3-m1 showing deteriorated roots and intact enamel; note that p4 and m1 show mild enamel hypoplasia. Scale bar: 20 mm.

**Table 1:** *Ceratotherium neumayri* (Osborn, 1900), Karacaşar, Late Vallesian of South Central Anatolia.

Length (estimated)	(590)
Maximum zygomatic width	(300)
Maximum frontal width	(190)
Palate width (at P2 level)	39
Palate width (at P4-M1 level)	86
Palate width (at M3 level)	81
Length of P2-M3 (left/right)	259/258
Length of P2-P4 (left/right)	124/126
Length of P3-P4 (left/right)	91/92
Length of M1-M3 (left/right)	156/154

Dimensions of the cranium HU-2011-1 (in mm).

The cranium was large and dolichocephalic (estimated length = 590; estimated width/length ratio ≈ 0.48). The preserved part of the nasal bones does not show any lateral apophysis. The *foramen infraorbitalis* is open above the middle of P4. The nasal notch reaches the anterior part of P4 while the anterior border of the orbit is located above the middle of M2. The nasal septum is not ossified. The nasal/lachrymal suture is not observable. The jugal/squamosal one, almost vanished, is straight and smooth. There are two *processi lacrymalis* but no lateral projection of the orbit. As estimated from the left half of the cranium, the zygomatic width/frontal width ratio appears to be slightly lesser than 1.50. There is no *processus postorbitalis* on the frontal. The base of the *processus zygomaticus maxillari* is high: it begins several centimetres above the neck of M2/3. The zygomatic arch is low and poorly developed. It forms a thin stripe transversely oblique (ca. 45°; diverging distally), with a faint *processus postorbitalis* on the jugal. The dorsal profile of the cranium is flat on the preserved part (Figure 3a). The external auditory pseudomeatus is partly closed, but the concerned area will necessitate further preparation. The thin and straight *hamulus pterygoideus* is very close to the M3. The posterior margin of the pterygoid is nearly horizontal. Morphological features hosted by the nasal, squamosal, parietal, and occipital bones are not observable, due to the incompleteness of the cranium. A low dome on the frontals attests to the presence of a small frontal horn. The fronto-parietal crests are smooth and widely separate (minimum distance ≈ 40 mm). The temporal fossa is hugely developed, forming a platform at each side of the braincase, which is still exaggerated by the obliquity of the zygomatic arches. In ventral view, the anterior start of the *processus zygomaticus maxillari* follows the curvature of the tooth row (Figure 3b). The palate is narrow, its width not exceeding 86 mm. The palatine fossa reaches the middle of M2. The shape and aspect of the articular tubercle for the mandible are not observable due to the temporo-mandibular junction of the specimen. The articular surface of the *processus postglenoidalis* defines a right dihedron in cross section. The *processus posttympanicus* is short. The *processus paraoccipitalis* is badly damaged and its total extent cannot be assessed (Figure 3a).

#### Mandible (Figures 3a–c; Table 2)

The tip of the symphysis is broken, but the preserved part is much upraised. The posterior margin of the symphysis reaches the middle of p3. Two *foramina mentalia* are located under the middle of p2 and p3, respectively. No *sulcus mylohyoideus* is observable, but the concerned area would need further preparation. The ventral border of the *corpus mandibulare* is much convex. The ramus is deep mesio-distally, inclined up- and backward, with a rounded and blunt *angulus mandibulare*. There is no *spatium retromolare*. The *processus coronoideus* is short (30 mm-long) and triangular in lateral view. The *foramen mandibulare* is located well below the teeth neck line.

**Table 2:** *Ceratotherium neumayri* (Osborn, 1900), Karacaşar, Late Vallesian of South Central Anatolia.

Length (preserved/estimated)	462/510
Length without symphysis	410
Height at p2-3	48
Height at p3-4	62
Height at p4-m1	75
Height at m1-2	85
Height at m2-3	84
height post m3	85
TD p2-3	59
TD p4-m1	−
TD m2-3	97
Width of symphysis	(63)
Width (external) under p3-4	(121)
Width (external) at the angulus	(205)
Height, *processus coronoideus*	(230)
Height at the articular tubercle	200
Length of p2-m3 (left)	272
Length of p2-p4 (left)	119
Length of p3-p4 (left)	86
Length of m1-m3 (left)	158

Dimensions of the mandible HU-2011-1 (in mm). All measurements taken from the left ramus, except when mentioned. TD, transverse diameter.

#### Dentition (Figures 3b–c, 4c–d, Table 3)

The dental formula is 3P-3M/3p-3m, as there is neither alveolus nor any trace of incisors, of canines, of D1/P1 on P2, or of d1/p1 on p2. The premolar series is long with respect to the molar series on upper teeth (L_P3–4_/L_M1–3_ ratio = 0.59); this ratio is lesser on lower teeth (L_p3–4_/L_m1–3_ ratio = 0.54), but still high at family level. There are no enamel foldings on the crowns. A thin layer of cement covers the ectoloph(id)s by places. The enamel is wrinkled at the neck and corrugated on the top of the crowns. The crowns are high but neither hypsodont nor subhypsodont (*sensu* [17]). The morphology of the roots is not observable.

**Table 3:** *Ceratotherium neumayri* (Osborn, 1900), Karacaşar, Late Vallesian of South Central Anatolia.

Specimen(l, left; r, right)	L		ant W		post W		H		
l P2		39		39		44		34	
l P3		48		58		54		47	
l P4		49		63		58		55	
l M1		58		63		62		50	
l M2		59		64		58		61	
l M3		51		59		Lect 59		58	
r P2		41		38		43		29	
r P3		50		56		49		42	
r P4		49		59		52		48	
r M1		58		61		−		−	
r M2		59		−		−		−	
r M3		50		58		Lect 58		−	
l p2		32		19		19		26	
l p3		43		26		29		32	
l p4		49		29		31		39	
l m1		52		29		34		32	
l m2		56		31		32		37	
l m3		55		29		27		>43	

Dental measurements of HU-2011-1 (in mm). ant., anterior; ect, ectoloph; H, height; L, length; post, posterior; W, width.

Faint traces of enamel hypoplasia are observable on several teeth of HU-2011-1, through contrasted dark/light horizontal strips on the protoloph of the right M3 (Figure 3b) and on the ectolophids of lower teeth. It is still more pronounced on the ectolophid of p4 and m1, at mid-crown height, with thinner enamel strips coinciding with *amelogenesis imperfecta* (Figure 4d).

#### Upper teeth

There is neither antecrochet nor anterior constriction of the protoloph on upper cheek teeth, except on M1 (Figures 3b, 4c). In occlusal view, the protoloph is straight on premolars and sigmoid on molars. The crochet and the crista are always present. Both are acute and sharp on premolars, joined only on P2 (medifossette) and equally developed on P2-3; the crochet is much more developed than the crista on P4-M3. The former is sagittal, with a small accessory tubercle lingual to it, while the crista is transversely oriented. The parastyle is sharp and sagittal. The paracone fold is present but weak and blunt. The metacone fold is absent from the whole series, except on P4, where it is restricted to the basal half of the teeth. A smooth mesostyle is present on P3-M2. The postfossette is deep and narrow on P2-M2.

#### Upper premolars

The premolars are molariform, with separate lingual cusps. There is no labial cingulum, but a strong lingual cingulum is always present (more developed in the mesial half of the tooth; Figure 3b). The metaloph is somewhat constricted on P2-3. On P2, the protocone is less developed than the hypocone. The protoloph is thin but continuous and connected with the ectoloph on P2-P4. The hypocone is posterior to the metacone on P2-4. There is no pseudometaloph on P3. P3 and P4 are tapering backwards in occlusal view (Table 3), at least at early wear stages, due to the sagittal obliquity of the ectoloph. A smooth mesostyle is observable on P3-4.

#### Upper molars

The molars are lacking a labial cingulum, while a lingual cingulum is present on M2-3 (restricted to the mesial part of the protocone on M2 and to a tubercle at the entrance of the median valley on M3). The metastyle is long. The metaloph is short – especially on M2; the posterior part of the ectoloph is concave on M2. There is no cristella and the posterior cingulum is low and reduced on upper molars. The metaloph is continuous and the central valley is open lingually) on M1-2. There is no lingual groove on the protocone of M2. The mesostyle is present but weak on M1 and M2. M3 has a triangular outline, with fused ectoloph and metaloph. The protoloph is transverse, even if curved backwards. There is no posterior groove on the ectometaloph. The distolingual cingulum is very smooth on M3, restricted to a couple of spurs separate by a shallow groove (Figure 3b), probably not homologous of the groove hollowed in the M3 of early rhinocerotids [17].

#### Lower teeth

There is neither labial, lingual cingulid, nor vertical roughness on lower cheek teeth, except on p4 (mesio-lingual cingulid ridge). The ectolophid groove is moderately deep, but developed until the neck (Figure 3c). The trigonid is angulous, especially on p2-m1, and it forms an acute angle on p2-m2 (right angle on m3). The metaconid and the entoconid are not constricted, with the exception of p4 (constricted entoconid). The posterior valley is open lingually and V-shaped in lingual view on all lower premolars. p2 has an individualised, spur-like, paralophid and a developed paraconid (Figure 3c). The hypolophid is oblique on lower molars. There is no lingual groove on the entoconid of m2-m3. The distal cingulid is extremely reduced on m3.

## Discussion

### Systematic Discussion

The presence of a smooth frontal boss and the absence of lower incisors in HU-2011-1 discard any referral to the most common rhinos recorded in the late Miocene of east Mediterranean, such as the hornless hippo-like aceratheres *Chilotherium* Ringström, 1924 and *Acerorhinus* Kretzoi, 1942 or the two-horned rhino *Stephanorhinus pikermiensis* (Toula, 1906). The presence of molariform premolars, of long and sagittally oriented crochets on upper premolars, and/or of flat ectolophs on upper cheek teeth impedes referring this skull as to the middle-sized rhinocerotine *Dicerorhinus schleiermacheri* (Kaup, 1832) or as to middle and late Miocene elasmotheriines [17].

On the other hand, most cranial, mandibular, and dental characters fit those observed in the large two-horned rhino “*Atelodus neumayri* Osborn, 1900”, here referred to as *Ceratotherium neumayri* (Osborn, 1900), as detailed in several works [25]–[27]. However, two *processi lacrymalis* are retained, there is no lateral projection of the orbit but a faint *processus postorbitalis* on the jugal, contrary to what is observed in the cranium from Akkasdağı (no *processus lacrymalis*; wide lateral projection of the orbit; no *processus postorbitalis* on the jugal [26]). The latter cranium is ca. 15% larger and it originates from younger deposits (ca. 7 Ma-old [5]). The protoloph is straight on premolars and sigmoid on molars while it is curved backwards on the whole upper series of *C. neumayri* from Akkasdağı [26]. The metaloph is somewhat constricted on P2-3 (unconstricted in Akkasdağı). There is a strong lingual cingulum on P2-4 (weak in Akkasdağı).

Other morphological features are consistent with the large two-horned rhino from the late Miocene of the Eastern Mediterranean Province [25]–[31], which leads us to consider it as an early representative of *C. neumayri*.

The generic assignment of “*Atelodus neumayri* Osborn, 1900”, within *Ceratotherium* Gray, 1868, *Diceros* Gray, 1821, or *Pliodiceros* Kretzoi, 1942, has been a matter of controversy for the last decades. This controversy is primarily due to contradictory i) dental similarities between the living black rhino (*Diceros bicornis*) and the late Miocene species we refer to as *Ceratotherium neumayri* here, and ii) cranial similarities between the latter taxon and the living white rhino (*Ceratotherium simum*). This topic is widely detailed by Hernesniemi et al. [32]. Accordingly, some authors consider the former generic assignment [32]–[34] while others go for the latter [25]–[27], [31], [35]. Moreover, the co-occurrence of several distinct lineages of two-horned rhinos in the late Miocene of Eurasia is highly suspected [31]–[32], [34]. Accordingly, and pending a formal phylogenetically-aimed revision of late Neogene two-horned rhinos – which is far beyond the scope of the current work –, we have chosen to use the most conservative binomen for our specimen, i.e. *Ceratotherium neumayri* [31].

### Taphonomy

Most parts of the premaxillae, nasals and parietals of HU-2011-1 are abraded and destroyed (Figure 3a). By contrast with the vertical erosion of the occipital part of the skull, obviously scalped by a recent stream of fluvial origin (Figure 2a-b), such damages appear to be tied to the transport of the skull before deposition, as the concerned areas, both salient and fragile, were found fully covered by ash-rich primary matrix. This interpretation is substantiated by the recovery of isolated bone fragments close to the skull, including a rib closely stuck to the naso-frontal roof of the cranium, i.e. south to it (Figure 3b), and unambiguously testifying to the presence of a unidirectional current of southern origin and flowing roughly to the North by the time of deposition of the Kavak-4 ignimbrite subunit.

In addition, the general aspect of HU-2011-1 is quite unusual for a fossil rhino skull, with a rough bone surface, brittle dentine, and broken or partly disintegrated roots (Figures 3–4). Given the volcaniclastic nature of the embedding matrix, it appeared legitimate to wonder if heat would be responsible for such dental and osseous structural changes or not. The effects of heating on bones and teeth have long been studied in forensic and archaeological perspectives; they consist in studying crystallographic changes, morphology, and shrinkage, even if most experimental and case studies focus on colour changes (for review, see [36]). Accordingly, we aimed at reconstructing the hypothetical conditions that would have led to the death of the rhino, and at proposing a plausible scenario for this event.

In the present case, due to changes through long-termed diagenesis, the colour of HU-2011-1 may not to be related to the initial heating, but to the burial process instead, tied to the presence of trace elements in the matrix (pale pinkish colour for bones and cement, as is the surrounding ignimbrite; olive green for enamel and medium brown for dentine).

**Figure 5:**
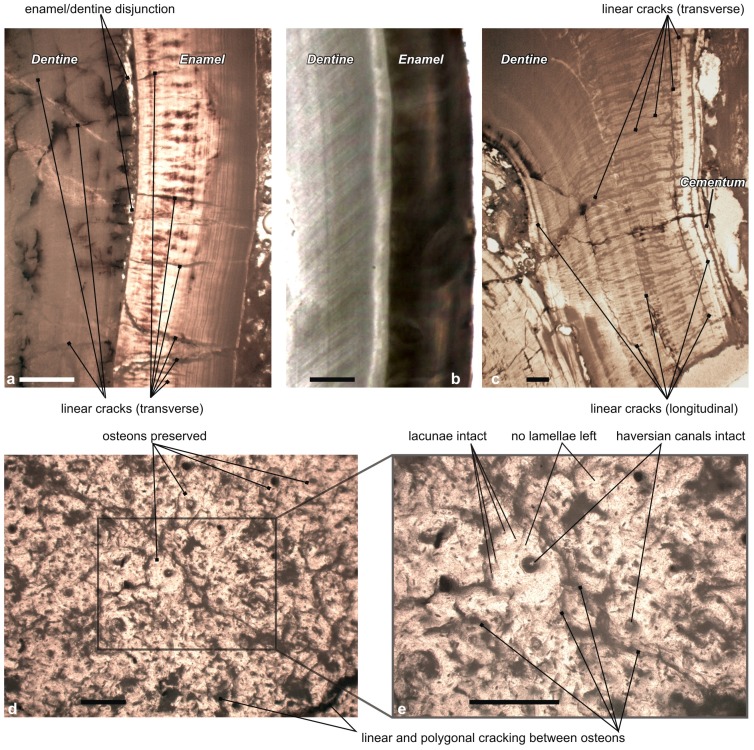
Heating effects on the Karacaşar rhino skull as revealed by light microphotographs of hard tissues. a. Detail of the crown of the right p3, with large cracks running through the dentine and oblique cracks affecting the enamel, separation of the enamel-dentine junction (secondarily re-crystallized) and dense crack network affecting both the enamel and dentine, indicating direct exposure to a ∼400–450°C heat (scale bar, 1.0 mm). b. Control rhino tooth (unheated), without structural change (scale bar, 1.0 mm). c. Detail of the root of the right p3, with cracks passing across the cementum and dentine, and longitudinal cracks within the dentine, pointing to a ∼450°C heating exposure (scale bar, 1.0 mm). d. Thermal modification of cortical compact bone (right nasal bone): presence of linear and polygonal cracking between osteons (scale bar, 0.2 mm). e. Same thin section as in (d), but magnified twice (scale bar, 0.1 mm). Osteons, osteocyte lacunae, and haversian canals are preserved. Lamellae and canaliculi have vanished, thus pointing to short and controlled exposures at ≥300–400°C [8].

We have monitored both gross structural features (Figure 4) and eventual microscopic structural changes (Figure 5). We have compared these features to the ones detailed in published forensic and archaeological studies, and then interpreted them with respect to the results focusing on bones [8], [37] or dental tissues (cementum, dentine, and enamel [21], [23], [38]).

#### Gross examination and interpretation of related phenomena (Table 4)

The entire surface of the cranium and mandible from Karacaşar is rough and covered by parallel cracks coinciding with the delimitation of osseous fibres. Shipman et al. [21] showed that the surface of heated bones was becoming rough and striated, with extensive cracking due to dehydration, especially in case of direct thermal exposure (see also [37]).

**Table 4:** Temperatures the Karacaşar rhino skull HU-2011-1 was probably exposed to, as hypothesized by gross and microscopic analysis of organic elements.

Proxies	Diagnostic Features	Heating Conditions	Literature
*Gross anatomy* (macroscopic observation)	Rough bone surface on cranium and mandible; presence of parallel crackson bones (dehydration); mouth wide open (post-mortem dehydration andmuscle contraction); roots partly disintegrated; bone not disintegrated	Direct exposure (≥400°C)	[8], [21], [36]–[38]
*Tooth crowns* (microscopy)	Enamel-dentine disjunction; short trajectory fractures through enamel and dentine; transverse and longitudinal linear cracks (enamel+dentine)	Gradual and continued or direct exposure (∼450°C)	[23], [39]–[40]
*Roots* (microscopy)	Short trajectory fractures through cementum and dentine; transverse and longitudinal linear cracks (cementum+dentine)	Gradual and continued or direct exposure (∼450°C)	[23], [39]–[40]
*Compact bone* (microscopy)	Dense linear and polygonal cracking; osteons, osteocyte lacunae, and haversian canals preserved; lamellae and canaliculi vanished	Short but controlled exposure (≥300–400°C)	[8], [21]

**Table 5:** Volcanic rocks likely to embed fossil vertebrates and associated morphological and/or taphonomic features, sorted by increasing temperature range.

Volcanic rocks	Associated Features on Fossils	Temperature Range	Literature
*Distal ashfalls* (Ashfall Beds, Akkasdağı)	Mouths closed on articulated skulls; connected skeletons; presence of scavenging traces; no evidence for heating	No heating	[44]–[45], [50]
*Pyroclastic density currents* (79 AD Plinian eruption of the Vesuvius)	Victims show ‘life-like’, ‘sleep-like’ or ‘impact-like’ postures; instantaneous death; ‘pugilistic attitude’ and limb contraction (drastic muscle dehydration); structural changes in hard tissues; no noticeable transport	250–600°C	[8]
*Basalt flows*	Usually, no preservation of fossils; Casts exceptionally preserved (‘Blue Lake Rhino’)	>900°C	[41]–[43]

Preservation of the Karacaşar rhino (submitted to a ∼400–450°C temperature) is most likely tied to a pyroclastic density current, reminiscent of that of Pompeii-Herculaneum-Oplontis, of Vesuvius origin (79 AD).

Disintegration of the roots on HU-2011-1 is either superficial/partial (Figure 4c; labial roots of lower cheek teeth) or deep/complete (Figure 4b; lingual roots of right P4-M2). The experiments led by Karkhanis [38] on isolated human teeth exposed to heat document a similar root/crown breakage and subsequent cementum/dentine disintegration into smaller brittle particles with exposure to temperatures exceeding 400°C. As the present teeth are much larger than their human counterparts, and as they may have benefitted from the buffering effect of soft tissues (skin and muscles), the disintegration of the concerned roots would have occurred at noticeably higher peri-mortem environmental temperatures [8].

Moreover, the mouth wide open of HU-2011-1 (26°; Figure 4) is here interpreted as due to reflex movements tied to dehydration and protein denaturation of soft tissues such as muscles, when they still protect the hard bony and dental tissues [23], [36]).

#### Microscopic observations (Table 4)

*Teeth*: Even though teeth can survive to indirect temperatures reaching 1100°C, direct heat exposure may affect or even destroy their structure at much lower temperatures [23]. If heat is intense and heating fast (>450°C; 10-10^2^s [39]), the evaporation of the organic components inside tooth crowns induces the separation of the enamel layer from the dentine, the latter being less mineralized than the former [23]. A similar dentine-enamel disjunction occurs when teeth are submitted to controlled heat (i.e., gradual and continued exposure [23]). Roots (dentine covered by cementum) are protected by soft tissues and sheltered by bones in normal conditions, while enamel is more often directly in contact with the heating source. Accordingly, with respect to cementum and enamel, dentine seems to be the most reliable tissue for i) recognizing incinerated teeth [22], and ii) estimating a temperature range the corresponding remains were exposed to [23].

Experiments on young and aged teeth submitted to a gradual and continued increment of temperature show the appearance of short trajectory fractures through enamel and dentine, running from the external surface towards the inside of the teeth crowns at temperatures exceeding 300°C (small teeth) or 450°C (larger teeth) [23], [40]. In similar conditions (>300°C), human tooth roots become rough-surfaced and deep fissures occur, while cementum eventually separates from dentine [38]. Such structural changes do not occur in case of direct heat exposures.

In thin sections of tooth crowns from the Karacaşar rhino, i) enamel and dentine are widely separate at the amelodentinal limit, ii) transverse and longitudinal linear cracks are visible in both dentine and enamel (Figure 5a), iii) several fractures run inward across the enamel and dentine layers (Figure 5a) or across both the cementum and dentine layers (Figure 5c). Such structural changes in dentine and enamel layers widely contrast with what can be observed on an unheated modern rhino tooth crown (Figure 5b). The shape and density of transverse cracks crossing all the hard tissues point to a continued exposure to the heating source, with temperatures around 450°C, following previous experiments [23], [39]–[40].

*Bone*: In thin sections, compact bone of the Karacaşar rhino skull shows both linear and polygonal crackings (Figure 5d) and its histological structure is only partly preserved: osteons, osteocyte lacunae, and haversian canals are intact, while lamellae and canaliculi have totally vanished with heat (Figure 5e). Given that the cortical layer of rhino bones is thicker than its counterpart in horses, it can be assumed that the Karacaşar rhino skull was exposed to temperatures around 400°C: compact horse bones exposed at temperatures of 200–300°C preserve their histological integrity, even if the concerned bones show linear microcracking between osteons [8]. At temperatures greater or equal to 285°C, distinctive cracking patterns develop in subchondral bones as a consequence of dehydration in horse and human bones [21]. Extreme polygonal cracking and partial vanishing of the lamellar structure first occur at 400°C while, at 500°C and above, only osteocyte lacunae are still visible, as canaliculi, osteons, and lamellae have completely vanished ([8]: figure 7). At higher temperatures, bone usually becomes highly friable and it disintegrates rapidly [8].

### Comparison to Similar Cases of Preservation within Volcanic Rocks (Table 5)

- Preservation of fossils in volcanic rocks, as exemplified by the Blue Lake rhino (Table 5): A rare case of fossil preservation within a basalt flow occurred 15–16 Ma ago in the Grand Coulee area, Washington, USA, when a complete rhinoceros body, floating upside down in a lake, was moulded within a cooling-down Columbia River basalt flood [41]–[43]. Generally, basalts flow at temperatures exceeding 900°C, which “would supposedly destroy the skin, flesh, and bones of an engulfed animal [41]. However, this exceptional case occurred in a pond or a lake, which led to the formation of instant-chilled “pillow lavas”, in turn allowing the preservation of a few phosphatized remains of the concerned rhino (left and right jaw and carpal bone [41], [43]). Such scenario does not coincide with i) the regional context as described in the late Miocene of the Nevşehir-Plateau [11]–[13] and ii) the temperatures the skull seems to have been exposed to (Table 4).
- Distal ashfalls provoke a delayed death (Table 5): Small ash particles are known to fallout thousand of kilometres away from the volcanic source and cause catastrophic death assemblages, as observed for the ∼11.8 Ma-old eruption of the Bruneau-Jarbridge caldera of SW Idaho, USA [44]–[45]. This eruption occurred at ∼116°W, and westerly prevailing winds propagated airborne particles more than 1400 km more to the East [45]. As a consequence, a 2 m-thick volcaniclastic ash layer deposited downwind in Antelope County, NE Nebraska (∼98°W) and buried a spectacular assemblage of 200 connected skeletons of a wide array of large mammals (rhinos, three-toed horses, camels, and deers) at Ashfall Fossil Beds, within a four-week estimated period [4], [46]–[48]. If some individuals died suddenly from suffocation, most of them survived the ashfall and experienced severe lung diseases before dying [49]. Regardless of their taxonomic affinities, all the specimens have their mouth closed (http://ashfall.unl.edu), which strikingly contrasts with the widely open mouth of the Karacaşar rhino (Figure 3). Moreover, the present specimen was disconnected from the corresponding postcranial skeleton (not recovered to date) before deposition, which further points to distinct peri-mortem histories.

The Akkaşdağı bone beds, also from the late Miocene of Central Anatolia, accumulated in a single flow of massive tuffs, rich in pumices and lithic clasts (7.1±0.1 Ma [5]). Both mortality profiles of the most abundant mammals in Akkaşdağı and scavenging traces on the corresponding remains point to a catastrophic event, most probably due to gas emanation tied to a volcanic eruption [50]. Even if their lithology and geochemical composition are comparable, the Akkaşdağı tuff and the Kavak flow do not originate from the same source area [49]. Moreover, the taphonomic processes having led to the fantastic vertebrate accumulation of Akkaşdağı widely differ from those that might have caused the preservation of the Karacaşar rhino, in that (i) water is considered as having been the main accumulating agent in the former locality and (ii) there is no evidence for heating effect on the>3200 inventoried specimens in Akkaşdağı [50].

- Pyroclastic density currents as documented by the 79 AD Vesuvius eruption (Table 5): The 79 AD Plinian eruption of the Vesuvius provoked instantaneous death of thousands humans and animals within a 15 km-wide area, i.e. in Pompeii, Herculaneum, and Oplontis, but pyroclastic ashes accumulated up to 20 km away from the vent [8]. This eruption allowed preservation of connected skeletons (or casts of the corresponding bodies). The temperature range commonly measured for similar pyroclastic flows is 250–600°C, which is in good agreement with computed estimates [51]. The compared analysis of human victim bones (showing linear and polygonal cracking but no neoformed features such as globules), of silverware (melt), and of wood objects and food (carbonized) points to a 250–300°C range in Pompeii (10 km away [8]). This temperature probably reached 500–800°C in Herculaneum and Oplontis, i.e. closer to the vent (5 and 7 km away, respectively [8]). As a result, a very short exposure to the pyroclastic surge (10 to 10^2^ seconds-long) was certainly lethal, the thermal effects of which caused instantaneous death up to 15 km away from the Vesuvius [8]. The correspondence is striking between the structural changes of hard tissues as observed in both the Karacaşar rhino skull and Pompeii-Herculaneum-Oplontis human and pet remains, which points to similar heating conditions in both events. The recovery of carbonized plant remains in the Kavak-4 subunit [12], [14], i.e. the very ignimbrite unit which yielded the rhino skull, further testifies to the match between the concerned events, as far as temperature is concerned. However, contrary to what is observed in Pompeii, Herculaneum, and Oplontis (no transport), the Karacaşar rhino skull was further transported and rolled northward within the channelized pyroclastic flow, as illustrated by the differential breakage of salient and fragile areas (Figure 3a).

### Proposed Scenario for the Preservation of the Karacaşar Rhino Skull

The rhino skull HU-2011-1 shows obvious stigmata of a continued exposure to heat. Baking mostly affected the aspect and preservation of dentine (occlusal surface and roots: brittle and disintegrated into small fragments; Figure 5) and of bone surface (Figure 4a, 4c), while enamel seems to be well preserved, at least in a macroscopic perspective, even though extremely fragile and fractured by places. The rough and corrugated aspect of the bony surface (Figure 4a, 4c) is primarily assumed to result from a continued exposure to warm volcaniclastics which constitute its matrix (Table 4).

A strikingly similar case of “baked” fossil mammal remains was reported in the early Oligocene of Chilean Andes [52]. It concerned pseudoglyptodont xenarthran specimens (a fossil relative of living armadillos) that “may have been engulfed in a lahar or volcanic debris flow and literally cooked to death, with the thinner parts of the cranium and jaws reduced to cinders and only the more massive parts remaining, more or less in their natural positions.” No other mention was made concerning the hypothetical conditions (temperature, density, or exposure time) that led to the selective decay of the xenarthran in the corresponding article, but it can be hypothesized that the taphonomic process was similar to what occurred in Karacaşar.

After examination and comparison of both gross and microscopic features of hard preserved tissues, it can be assumed that a pyroclastic flow, somewhat similar to the one tied to the Plinian eruption of the Vesuvius in 79 AD, has occurred 9.2±0.1 Ma ago in what is today Cappadocia (Tables 4–5). A pyroclastic density current sourced from the Çardak caldera (Figure 1) most likely i) provoked the instant death of the Karacaşar rhino, before the body of the latter ii) experienced severe dehydration (leading to the wide and sustainable opening of the mouth), iii) was then dismembered within the pyroclastic flow of subaerial origin, the skull being separated from the remnant body and baked under a temperature approximating 400°C, iv) then transported northward, rolled, and trapped in disarray into that pyroclastic flow forming the pinkish Kavak-4 ignimbrite, and v) was incidentally found by four of us in 2010, ∼30 km North from the upper Miocene vent.

## References

[1] Briggs DEG, Siveter DJ, Siveter DJ (1996) Soft-bodied fossils from a Silurian volcaniclastic deposit. Nature 382: 248–250.

[2] Leakey MD, Hay RL (1979) Pliocene footprints in the Laetolil Beds at Laetoli, northern Tanzania. Nature 78: 317–323.

[3] White TD, Suwa G (1987) Hominid footprints at Laetoli: facts and interpretations. Amer J Phys Anthrop 72: 485–514. 3111270 10.1002/ajpa.1330720409

[4] Voorhies MR, Thomasson JR (1979) Fossil grass anthoecia within Miocene rhinoceros skeletons: diet in an extinct species. Science 206: 331–333. 17733681 10.1126/science.206.4416.331

[5] Karadenizli L, Seyitoglu G, Sen S, Arnaud N, Kazanci N, et al (2005) Mammal bearing late Miocene tuffs of the Akkasdagi region; distribution, age, petrographical and geochemical characteristics. Geodiversitas 27: 553–566.

[6] Branney MJ, Kokelaar BP (2002) Pyroclastic Density Currents and the Sedimentation of Ignimbrites. Geol Soc London Mem 27, 1–143.

[7] Baxter PJ (1990) Medical effects of volcanic eruptions, I. Main causes of death and injury. Bull Volcanol 52: 532–544.

[8] Mastrolorenzo G, Petrone P, Pappalardo L, Guarino FM (2010) Lethal Thermal Impact at Periphery of Pyroclastic Surges: Evidences at Pompeii. PLoS ONE 5(6): e11127 doi:10.1371/journal.pone.0011127 Accessed 25 September 2011. 20559555 10.1371/journal.pone.0011127 PMC2886100

[9] Behrensmeyer AK (2007) Bonebeds through geologic time. In: Rogers R, Eberth D, Fiorillo T, editors. Bonebeds: Genesis, Analysis, and Paleobiological Significance. Chicago: University of Chicago Press, pp. 65–102.

[10] Mues-Schumacher U, Schumacher R (1996) Problems of stratigraphic correlation and new K–Ar data for ignimbrites from Cappadocia, Central Turkey. Int Geol Rev 38: 737–746.

[11] Le Pennec JL, Temel A, Froger JL, Sen S, Gourgaud A, et al (2005) Stratigraphy and age of the Cappadocia ignimbrites, Turkey: reconciling field constraints with paleontologic, radiochronologic, geochemical and paleomagnetic data. J Volcanol Geotherm Res 141: 45–64.

[12] Aydar E, Schmitt AK, Çubukçu HE, Akin L, Ersoy O, et al (2012) Correlation of ignimbrites in the central Anatolian volcanic province using zircon and plagioclase ages and zircon compositions. J Volcanol Geotherm Res 213–214: 83–97.

[13] Froger JL, Lénat JF, Chorowicz J, Le Pennec JL, Bourdier JL, et al (1998) Hidden calderas evidenced by multisource geophysical data; example of Cappadocian calderas, Central Anatolia. J Volcanol Geotherm Res 185: 99–128.

[14] Le Pennec JL, Bourdier JL, Froger JL, Temel A, Camus G, et al (1994) Neogene ignimbrites of the Nevsehir Plateau (Central Turkey), stratigraphy, distribution and source constraints. J Volcanol Geotherm Res 63: 59–87.

[15] Guérin C (1980) Les Rhinocéros (Mammalia, Perissodactyla) du Miocène terminal au Pléistocène supérieur en Europe occidentale. Comparaison avec les espèces actuelles. Doc Lab Géol Univ Lyon, Sci Terre 79: 1–1184.

[16] Heissig K (1969) Die Rhinocerotidae (Mammalia) aus der oberoligozänen Spaltenfüllung von Gaimersheim. Abh Bayer Akad Wiss, Math-Naturwiss Kl, NF 138: 1–133.

[17] Antoine PO (2002) Phylogénie et évolution des Elasmotheriina (Mammalia, Rhinocerotidae). Mém Mus natl Hist nat 188: 1–359.

[18] Antoine PO (2003) Middle Miocene elasmotheriine Rhinocerotidae from China and Mongolia: taxonomic revision and phylogenetic relationships. Zool Scripta 32: 95–118.

[19] Antoine PO, Duranthon F, Welcomme JL (2003) *Alicornops* (Mammalia, Rhinocerotidae) dans le Miocène supérieur des Collines Bugti (Balouchistan, Pakistan): implications phylogénétiques. Geodiversitas 25: 575–603.

[20] Antoine PO, Downing KF, Crochet JY, Duranthon F, Flynn LJ, et al (2010) A revision of *Aceratherium blanfordi* Lydekker, 1884 (Mammalia: Rhinocerotidae) from the early Miocene of Pakistan: postcranials as a key. Zool J Linn Soc 160: 139–194.

[21] Shipman P, Foster G, Schoeninger M (1984) Burnt bones and teeth: an experimental study of color, morphology, crystal structure and shrinkage. J Archaeol Sci 11: 307–325.

[22] Myers SL, Williams JM, Hodges JS (1999) Effects of Extreme Heat on Teeth with Implications for Histological Processing. J Forensic Sci 44: 805–809. 10432614

[23] Fereira JL, Espina de Fereira A, Ortega AI (2008) Methods for the Analysis of Hard Dental Tissues Exposed to High Temperatures. Forensic Sci Int 178: 119–124. 18434052 10.1016/j.forsciint.2007.12.009

[24] Hillman-Smith AKK, Owen-Smith N, Anderson JL, Hall-Martin AJ, Selaladi JP (1986) Age estimation of the White rhinoceros (*Ceratotherium simum*). J Zool 210: 355–379.

[25] Geraads D (1988) Révision des Rhinocerotinae (Mammalia) du Turolien de Pikermi. Comparaison avec les espèces voisines. Ann Paléont 74: 13–41.

[26] Antoine PO, Saraç G (2005) The late Miocene mammalian locality of Akkasdagi, Turkey: Rhinocerotidae. Geodiversitas 27: 601–632.

[27] Giaourtsakis I, Theodorou G, Roussiakis S, Athanassiou A, Iliopoulos G (2006) Late Miocene horned rhinoceroses (Rhinocerotinae, Mammalia) from Kerassia (Euboea, Greece). N Jb Geol Paläont Abh 239: 367–398.

[28] Heissig K (1975a) Rhinocerotidae aus dem Jungtertiär Anatoliens. Geol Jb B 15: 145–151.

[29] Heissig K (1975b) Rhinocerotidae aus dem Jungtertiär Anatoliens. University of Munich. 600 p. Unpublished Manuscript.

[30] Heissig K (1996) The stratigraphical range of fossil rhinoceroses in the Late Neogene of Europe and the Eastern Mediterranean. In Bernor RL, Fahlbusch V, Mittmann HW, editors. The Evolution of Western Eurasian Neogene Mammal Faunas. New York: Columbia University Press, pp. 339–347.

[31] Fortelius M, Heissig K, Saraç G, Sen S (2003) Rhinocerotidae (Perissodactyla). In Fortelius M, Kappelman J, Sen S, Bernor RL, editors. Geology and Paleontology of the Miocene Sinap Formation, Turkey. New York: Columbia University Press, pp. 282–307.

[32] Hernesniemi E, Giaourtsakis IX, Evans AR, Fortelius M (2011) Rhinocerotidae. In Harrison T, editor. Paleontology and Geology of Laetoli: Human Evolution in Context. Volume 2: Fossil Hominins and the Associated Fauna, Vertebrate Paleobiology and Paleoanthropology. Houten: Springer Science+Business Media, pp. 275–294. DOI 10.1007/978-90-481-9962-4-11.

[33] Giaourtsakis IX (2009) The Late Miocene Mammal Faunas of the Mytilinii Basin, Samos Island, Grece: New Collection. 9. Rhinocerotidae. Beitr Paläont 31: 157–187.

[34] Giaourtsakis I, Pehlevan C, Haile-Selassie Y (2009) Rhinocerotidae. In Haile-Selassie Y & WoldeGabriel G, editors. *Ardipithicus kabbada*: Late Miocene evidence from the Middle Awash, Ethiopia. Berkeley: University of California Press, pp. 429–468.

[35] Geraads D (2005) Pliocene Rhinocerotidae (Mammalia) from Hadar and Dikika (Lower Awash, Ethiopia), and a revision of the origin of modern African rhinos. J Vert Paleont 25: 451–461.

[36] Ubelaker DH (2009) The Forensic Evaluation of Burned Skeletal Remains: A Synthesis. Forensic Sci Int 183: 1–5. 19010619 10.1016/j.forsciint.2008.09.019

[37] Pijoan CM, Mansilla J, Leboreiro I, Lara VH, Bosch P (2007) Thermal Alterations in Archaeological Bones. Archaeometry 49: 713–727.

[38] Karkhanis S (2008) Macroscopic and microscopic changes in incinerated deciduous teeth. Univ Western Australia. Unpublished MSc Thesis. 22785093

[39] Muller M, Bertrand MF, Quatrehomme G, Bolla M, Rocca JP (1998) Macroscopic and Microscopic Aspects of Incinerated Teeth. J Forensic Odont 16: 1–7. 9922754

[40] Savio C, Merlati G, Danesino P, Fassina G, Menghini P (2006) Radiographic Evaluation of Teeth Subjected to High Temperatures: Experimental Study to Aid Identification Processes. Forensic Sci Int 158: 108–116. 15993019 10.1016/j.forsciint.2005.05.003

[41] Chappell WM, Durham JW, Savage DE (1951) Mold of a rhinoceros in basalt, Lower Grand Coulee, Washington. GSA Bull 62: 907–918.

[42] Kaler KL (1988) The Blue Lake Rhinoceros. Washington Geol Newsletter 16: 3–8.

[43] Prothero DR (2005) The Evolution of North American rhinoceroses. Cambridge: Cambridge University Press, 218 p.

[44] Perkins ME, Brown FH, Nash WP, McIntosh W, Williams SK (1998) Sequence, age, and source of silicic fallout tuffs in middle to late Miocene basins of the northern Basin and Range province. GSA Bull 110: 344–360.

[45] Rose WI, Riley CM, Dartevelle S (2003) Sizes and Shapes of 10-Ma Distal Fall Pyroclasts in the Ogallala Group, Nebraska. J Geol 111: 115–124.

[46] Mead AJ (2000) Sexual dimorphism and paleoecology in *Teleoceras*, a North American Miocene rhinoceros. Paleobiology 26: 689–706.

[47] Mihlbachler MC (2003) Demography of Late Miocene Rhinoceroses (*Teleoceras proterum* and *Aphelops malacorhinus*) from Florida: linking mortality patterns and sociality in fossil assemblages. Paleobiology 29: 413–429.

[48] Famoso NA, Pagnac D (2011) A Comparison of the Clarendonian Equid Assemblages from the Mission Pit, South Dakota and Ashfall Fossil Beds, Nebraska. Trans Nebraska Acad Sci Affil Soc, 9. Available: http://digitalcommons.unl.edu/tnas/9.

[49] Beck DK (1995) Hypertrophic pulmonary osteodystrophy recognized in *Teleoceras major* (Mammalia: Rhinocerotidae) from the late Miocene of Nebraska. GSA Abstr 27: 38.

[50] Valli AMF (2005) Taphonomy of the late Miocene of Akkasdagi, Turkey. Geodiversitas 27: 793–808.

[51] Esposti Ongaro T, Neri A, Todesco M, Macedonio G (2002) Pyroclastic flow hazard assessment at Vesuvius by using numerical modelling. 2. Analysis of flow variables. Bull Volcanol 64: 178–191.

[52] McKenna MC, Wyss AR, Flynn JJ (2006) Paleogene pseudoglyptodont xenarthrans from central Chile and Argentine Patagonia. Amer Mus Novit 3536: 1–18.

